# Dietary Stilbenes May Mitigate Risk of Lung Cancer: Evidence from Two Prospective Cohort Studies in the UK and Japan

**DOI:** 10.3390/antiox15050563

**Published:** 2026-04-29

**Authors:** Xiaona Na, Shirai Kokoro, Keyang Liu, Zhihui Li, Akiko Tamakoshi, Ai Zhao, Hiroyasu Iso

**Affiliations:** 1 Vanke School of Public Health, Tsinghua University, Beijing 100084, China; nxn21@mails.tsinghua.edu.cn (X.N.); zhihuili@tsinghua.edu.cn (Z.L.); 2 Institute for Healthy China, Tsinghua University, Beijing 100084, China; 3 Department of Social Medicine and Behavioral Science, Division of Health Science, Graduate School of Medicine, Osaka University, Osaka 565-0871, Japan; shirai@sahs.med.osaka-u.ac.jp (S.K.); ky@sahs.med.osaka-u.ac.jp (K.L.); 4 Department of Clinical Epidemiology and Biostatistics, Children’s Hospital of Chongqing Medical University, National Clinical Research Center for Children and Adolescents’ Health and Diseases, Ministry of Education Key Laboratory of Child Development and Disorders, Chongqing Key Laboratory of Pediatric Metabolism and Inflammatory Diseases, Chongqing 400014, China; 5 Department of Public Health, Graduate School of Medicine, Hokkaido University, Sapporo 060-0815, Japan; tamaa@med.hokudai.ac.jp; 6 Institute for Global Health Policy Research, Bureau of International Cooperation, National Center for Global Health and Medicine, Tokyo 162-8655, Japan

**Keywords:** polyphenols, stilbenes, flavonoids, phenolic acids, lung cancer, cohort

## Abstract

(1) Background: Polyphenols are believed to prevent the development of various diseases by counteracting inflammation and oxidative stress. However, polyphenols are difficult to quantify in foods and show substantial variability in intake and epidemiological evidence on their relationship with lung cancer remains limited. This study explored the association between dietary polyphenols and lung cancer in two prospective cohorts. (2) Methods: Data from the UK Biobank and the Japan Collaborative Cohort Study for Evaluation of Cancer Risk (JACC) Study were used. The Phenol-Explorer^®^ database was used to assess the total and subtypes of dietary polyphenols. Lung cancer cases were identified using *The International Classification of Diseases, 10th revision*. Cox proportional hazards models were employed to estimate the association by hazard ratios (*HR*s) and 95% confidence intervals (*CI*s) among overall participants and in stratified analysis by smoking status and sex, which were further meta-analyzed. The associations of polyphenol compounds and food sources with lung cancer were also examined. (3) Results: A total of 177,971 and 57,971 participants from the UK Biobank and the JACC Study, respectively, were included. Wines and fruits were identified as the primary dietary sources of stilbenes. Dietary stilbenes were associated with a lower risk of lung cancer in fully adjusted models for both UK Biobank (*HR*: 0.849, 95% *CI*: 0.723–0.996) and JACC Study (*HR*: 0.803, 95% *CI*: 0.648–0.996), as well as in the meta-analysis (*HR*: 0.832, 95% *CI*: 0.732–0.946). (4) Conclusions: Considering pharmacokinetic properties and biological plausibility, our findings suggest that stilbenes may serve as markers of polyphenol-rich foods lowering lung cancer risk. Further biomarker-based and interventional studies are warranted to clarify causality and elucidate the underlying mechanisms.

## 1. Introduction

The global burden of lung cancer remains a significant public health challenge, accounting for 19.4% of all cancer-related deaths [1]. Lung cancer is highly prevalent, with an estimated 2.2 million new cases (11.4%) and 1.8 million deaths (18.0%) worldwide in 2020, making it the leading cause of cancer mortality in men and the second leading cause in women [2]. This pattern is also evident in certain developed countries, where lung cancer is the leading cause of death [2]. Globally, it also imposes the greatest economic burden among all cancers, with an estimated cost of USD 3.9 trillion [3]. Therefore, identifying modifiable risk factors to develop preventive strategies and reduce the burden of lung cancer is crucial.

According to the World Health Organization, lung cancer remains one of the leading causes of cancer-related mortality worldwide. Cigarette smoking is the predominant risk factor for lung cancer, accounting for the majority of cases [4]. In addition, exposure to secondhand smoke, ambient air pollution, occupational carcinogens, and genetic susceptibility are well-established contributors to lung carcinogenesis [4,5]. Despite the strong influence of smoking, at least one-third of all cancer cases could be preventable through dietary and lifestyle modifications [6,7]. Emerging evidence suggests that various factors, including sociodemographic characteristics, diet, and lifestyles, are associated with incidence of lung cancer. Epidemiological and experimental studies have indicated that higher intakes of vegetables and fruits, as well as moderate wine consumption, may help prevent lung cancer, likely due to the presence of antioxidants such as vitamin A, C and E and certain phytochemicals such as polyphenols, which act as chemo-preventive agents by delaying the progression of lung cancer through their anti-inflammatory activities, along with other components like fiber [8,9,10,11,12]. However, the components within these foods and beverages that contribute to lung cancer prevention have not been clearly identified in either epidemiological studies or randomized controlled trials [13]. Understanding which components play a role in lung cancer prevention may pave the way to lung cancer prevention and have significant public health implications.

Among these components, polyphenols, a significant group of naturally occurring phytochemicals, have demonstrated strong antioxidant properties in mechanistic studies [14]. In experimental models of cancers, polyphenols have been shown to inhibit key inflammatory signaling pathways such as nuclear factor-κB (NF-κB), suppress the expression of cyclooxygenase-2 (COX-2) and inducible nitric oxide synthase (iNOS), and reduce pro-inflammatory cytokines including tumor necrosis factor-α (TNF-α), interleukin-6 (IL-6), and interleukin-1β (IL-1β) [15,16,17]. However, their association with lung cancer has not been examined in epidemiological studies, and most of the mechanistic studies have focused on their therapeutic potential rather than their preventive effects; for example, polyphenols have been used as agents to treat lung cancer in recent studies [14]. However, the bioavailability of polyphenols is generally low and can vary substantially across different compounds and dietary sources. For example, only relatively large single doses (tens of milligrams or more) produce micromolar plasma concentrations of metabolites [18]. Moreover, their bioavailability varies considerably across compounds and dietary sources, and biomarker-based quantitative assessments are scarce, highlighting the need for further research to accurately assess the relationship between dietary polyphenols and lung cancer risk.

Therefore, in large-scale prospective studies, we aimed to explore the preventive effects of total dietary polyphenols and their subtypes on lung cancer using two national prospective studies in the UK and Japan. The inclusion of two national cohorts from the UK and Japan was intentional and scientifically motivated. These populations represent distinct Western and Eastern dietary patterns, resulting in markedly different polyphenol intake levels and food sources. This diversity in exposure profiles allows us to evaluate whether potential associations are consistent across different cultural and dietary contexts. Since smoking is the leading cause of lung cancer, contributing to oxidative stress, inflammation, and lung structure disruption, it is crucial to examine this association in both ever-smokers and never-smokers to determine whether polyphenols can counteract smoking-related lung cancer [13,19]. Additionally, considering the known sex differences in lung cancer susceptibility and risk factors [20,21,22], sex-stratified analysis was also conducted to explore potential heterogeneity. We also estimated the association of specific polyphenol compounds with lung cancer to identify which compounds may play potential roles in lung cancer prevention. Finally, we examined the association between polyphenol-containing foods and lung cancer to provide practical implications and public health recommendations.

## 2. Materials and Methods

### 2.1. Ethnical Approval

The UKB study was approved by the National Information Governance Board for Health and Social Care in England and Wales, the Community Health Index Advisory Group in Scotland, and the North West Multi-centre Research Ethics Committee (11/NW/0382). All participants gave written informed consent. Our study was conducted in accordance with the Declarations of Helsinki. Our access to data from the UKB cohort was approved by the UKB Ethics Advisory Committee (Application ID: 91486).

The JACC Study protocol was approved by the ethics committees of Hokkaido University, Nagoya University, and Osaka University. Informed written consent was provided from the participants themselves or the community leaders.

### 2.2. Study Design and Population

**Figure 1:**
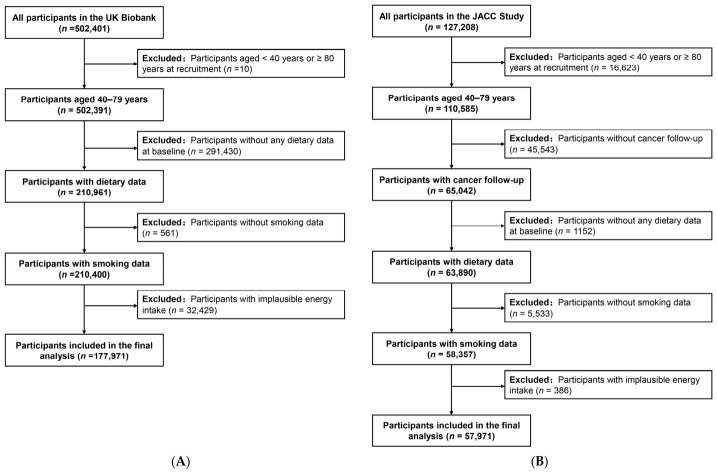
Flow chart in (**A**) UK Biobank and (**B**) JACC Study.

UK Biobank involved 500,000 participants aged 39 to 72 years from 22 assessment centers across England, Scotland, and Wales, recruited between 2006 and 2010 [22]. Further information about UK Biobank can be found online (http://www.ukbiobank.ac.uk). For this study, out of 502,401 participants, those aged <40 years or ≥80 years (*n* = 11), without data on dietary information (*n* = 291,430), and without smoking data (*n* = 566) were initially excluded. Participants with any cancer at baseline (*n* = 29,065) and implausible energy intake (*n* = 3358) were also excluded, resulting in a final sample of 177,971 participants for analysis (Figure 1A). The age-based exclusion was applied to harmonize the eligible age ranges between the two cohorts, ensuring comparability of the study populations. Implausible energy intake was defined as daily energy intake outside the range of 500–4000 kcal/day for women and 800–4500 kcal/day for men, in line with standard nutritional epidemiology practices [23].

The JACC Study, initiated between 1988 and 1990, recruited 127,208 Japanese individuals aged ≥40 years from 45 district areas [24]. Follow-up on cancer incidence was conducted until the end of 2009 in 24 of these areas. Further information can be found elsewhere [25]. In this study, following the same exclusion criteria as the UK Biobank and additionally excluding participants without cancer follow-up data, 57,971 participants were included (Figure 1B).

### 2.3. Assessments of Food Intakes and Dietary Polyphenols

Food and beverage intakes were assessed using 24 h dietary recalls in UK Biobank and a semi-quantitative food frequency questionnaire (FFQ) in the JACC Study. In UK Biobank, participants were invited to complete the dietary survey on five occasions in 2009–2012. Dietary data were collected using the Oxford WebQ (http://www.ceu.ox.ac.uk/research/oxford-webq [accessed on 14 August 2025]) in UK Biobank, a web-based assessment tool that evaluates the quantity/volumes of foods and beverages consumed in the past 24 h. Validation studies have confirmed its reliability [26]. In this study, participants with at least one dietary survey were included, and average food intake was calculated for those who completed two or more dietary surveys. Daily food and beverage intake quantities were derived by dividing the total consumption reported in these surveys by the number of dietary surveys completed. Energy intake was calculated using McCance and Widdowson’s *Composition of Food, 5th edition* [27].

In the JACC Study, FFQ was used to assess the intake of 40 food items over the previous 12 months at baseline, with frequency options ranging from “seldom” to “almost every day” [24]. Portion size for each food was determined and validated using four 3-day weighed dietary records over a one-year period [28]. The same predefined portion sizes were applied uniformly to ensure comparability across the 45 districts. Daily food and beverage intake quantities were calculated by dividing the total monthly intake (frequency multiplied by portion size) by 30. *The Japan Food Tables (5th Edition)* was used to calculate energy intake [29].

Participants without completed baseline dietary questionnaires were excluded. Within completed assessments, unreported food items were considered as non-consumed, and no additional imputation was performed. Dietary polyphenols at baseline were assessed using the Phenol-Explorer^®^ database (www.phenol-explorer.eu), the first comprehensive database on polyphenol content in foods, containing 500 different polyphenols in over 400 foods. This database also considers the cooking and processing of foods via retention factors, and we directly used these values in our calculations [30]. The content of polyphenols was expressed in mg/100 g of food fresh weight. In this study, according to the Phenol-Explorer^®^ database, foods containing polyphenols were all included, and those not containing any polyphenols, such as meat, were not considered for the calculation. For each participant, individual food items were first coded according to standard food codes, and were linked to the Phenol-Explorer^®^ database. Unreported foods were considered as not consumed when calculating dietary polyphenol intake, while participants with completely missing or implausible dietary data were excluded. Daily actual intake quantities for five subtypes of dietary polyphenols were calculated by multiplying the consumed amount of each food item by its polyphenol content and summing across all food items: flavonoids, phenolic acids, lignans, stilbenes, and other polyphenols. Other polyphenols mainly comprise simple phenols (e.g., tyrosol and hydroxytyrosol), alkylphenols (e.g., alkylresorcinols), and other minor polyphenolic compounds. For further analysis, quantities of dietary polyphenols were categorized into three groups by their tertiles.

### 2.4. Lung Cancer Ascertainment

The outcome was incident lung cancer, determined through linkage to national health records and population-based cancer registries. In UK Biobank, the dates and causes of hospitalizations were identified via record linkage to Health Episode Statistics (England and Wales) and the Scottish Morbidity Records (Scotland). Hospital admission data were available until September 2021 in England, July 2021 in Scotland and February 2018 in Wales. For the JACC Study, cancer incidence was determined using population-based cancer registries or major hospital records. In some areas out of 24 towns with cancer follow-up data, cancer follow-up ended earlier (one, four, one, one, eight, one, two, and two areas by the end of 1994, 1997, 1999, 2000, 2002, 2003, 2006, and 2008, respectively).

Lung cancer was identified using *The International Classification of Diseases, 10th revision* (ICD-10) as C34. Any cancer-at-baseline exclusions were identified by ICD-10 as C00–D48 and self-report [31]. The date of diagnosis was defined as the registry-confirmed diagnosis date; if unavailable, the earliest clinically recorded diagnosis date was used. Throughout the follow-up, each participant was censored at the first incidence of lung cancer, death, loss to follow-up, or the end of follow-up, whichever occurred first. Case ascertainment was not restricted to death-certificate-only cases; death records were used solely to determine vital status and censoring during follow-up.

### 2.5. Covariates

The covariates included the following categories: (1) Sociodemographic characteristics: age, sex, ethnicity (only in UK Biobank), educational level, socioeconomic deprivation status measured using Townsend deprivation index (TDI) (only in UK Biobank), and marital status (only in JACC Study); (2) Lifestyle characteristics: active and passive smoking, physical activity, sleep duration, BMI, and family history of lung cancer; (3) Dietary information: energy intake and supplementation; and (4) Psychological condition: depressive and anxiety disorders in UK Biobank and perceived stress measured by self-reported item in JACC Study.

### 2.6. Statistical Analyses

All statistical analyses were performed by R software version 4.4.0 (R Development Core Team, Vienna, Austria). A *p*-value of <0.05 (two-tailed) was considered statistically significant. As this analysis involved multiple compounds without pre-specified hypotheses, formal multiplicity corrections were not applied. This study is therefore considered exploratory. Before the analysis, multiple imputation by chained equations was conducted using the *mice* package (version 3.16.0) to impute missing covariate values, assuming that data were randomly missing [32]. Five datasets were generated through the 100th iteration using the random forest method.

Descriptive statistics were used to describe characteristics across the two cohorts. For continuous variables, *mean* (*SD*) and *median* (lower quartile [*Q*_1_], upper quartile [*Q*_3_]) were used to present normally distributed variables and skewed variables, respectively. Frequency (%) was used to describe categorical variables.

**Figure A1:**
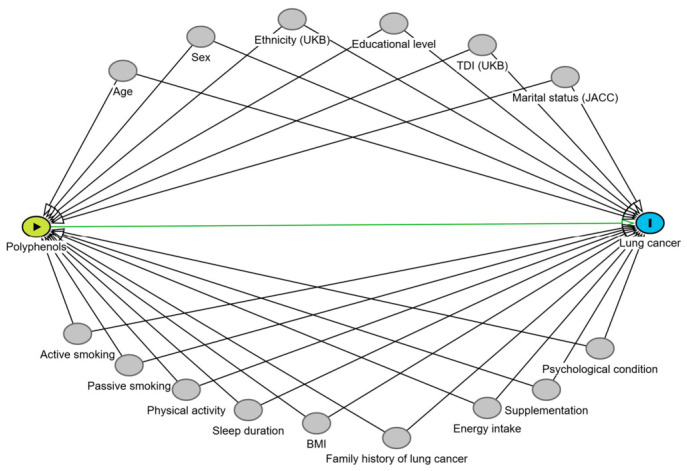
Directed acyclic diagram of analysis for dietary polyphenols and lung cancer. Abbreviations: UKB, UK Biobank; JACC, Japan Collaborative Cohort Study for Evaluation of Cancer Risk; TDI, Townsend deprivation index; BMI, body mass index.

Cox proportional hazards models were performed using *survival* (version 3.5-7) and *survminer* (version 0.4.9) packages to estimate the association between dietary polyphenols and incident lung cancer, with hazard ratio (*HR*) and 95% confidence interval (*CI*) as effect sizes. Four models were constructed: an unadjusted model; Model 1 adjusted for age (continuous), sex (male/female), ethnicity (only in the UK Biobank, White/Asian/Black/Others), educational level (primary or below/secondary/university), Townsend deprivation index (only in the UK Biobank, continuous), and marital status (only in the JACC Study, married/bereaved/divorced/single); Model 2 further adjusted for active and passive smoking status (never/ever), physical activity (low/moderate/high level of International Physical Activity Questionnaire in UK Biobank, almost never/1–2 h weekly/>2 h weekly of sports frequency in JACC Study), sleep duration (normal/shorter/longer), overweight and obesity status defined by BMI categories in their countries, and family history of lung cancer (yes/no); Model 3 further adjusted for energy intake (continuous), supplementation (yes/no), and psychological condition (depressive and anxiety disorders [yes/no] in UK Biobank, perceived stress [yes/no] in JACC Study). The specific analysis strategy has been presented as a directed acyclic diagram (Figure A1). To examine the effects of dietary polyphenols in different subgroups, stratified analyses of Cox proportional hazards models by smoking status (ever-smokers and never-smokers) and sex (males/females) were conducted. We further analyzed the association within four subgroups defined by both smoking status and sex. The above results for the Cox proportional hazards model with full adjustment among overall participants and subgroups from the individual study were meta-analyzed using either a fixed- or random-effects model by *meta* package (version 7.0-0), depending on the level of heterogeneity. Specifically, a fixed-effects model was applied when I^2^ < 50%, and a random-effects model was used when I^2^ ≥ 50%.

Based on the results of Cox proportional hazards models, we further explored the association of each compound in stilbenes with lung cancer in the two cohorts. Given the limited number of participants consuming these compounds, they were categorized into two groups according to median intake quantities. For compounds with a median intake of zero, participants were categorized as non-consumers (intake = 0) versus consumers (intake > 0). In both cohorts, the proportion of participants with zero intake varied by compound, ranging from approximately 10% to 30% in the UK Biobank and 40% to 60% in the JACC Study.

Finally, a food-based analysis was conducted using the Cox proportional hazards model, adjusting for the same covariates as in the main analysis. Food intakes were categorized into two groups: by median for frequently consumed foods and by consumption status (yes/no) for infrequently consumed foods.

We also conducted several sensitivity analyses. First, we performed a landmark analysis, restricting the main analysis to participants with over two years of follow-up to avoid reverse causality. Second, we repeated the analysis without imputing missing covariate values. Lastly, we estimated the association between average polyphenol intake and lung cancer among participants with at least two dietary surveys on five occasions in the UK Biobank to assess the stability of our results.

## 3. Results

Over a median (*Q*_1_, *Q*_3_) follow-up of 13.18 (12.60, 13.98) years and 13.33 (7.46, 18.03) years, 995 (0.6%) and 610 (1.1%) participants developed lung cancer in the UK Biobank and the JACC Study, respectively.

The basic characteristics of the participants in the two cohorts are presented in Table 1. While the baseline age was similar, other characteristics differed. Compared to the UK Biobank participants, those in the JACC Study were more likely to be female and have lower educational levels. Additionally, JACC Study participants had a lower proportion of active ever-smokers but higher passive ever-smokers, lower physical activity, shorter sleep duration, lower BMI, and a higher family history of lung cancer. They also had lower energy intake and were less likely to take supplements. For all variables that were directly comparable between the two cohorts, formal statistical tests were performed, and the differences were statistically significant (all *p* < 0.001).

**Figure 2:**
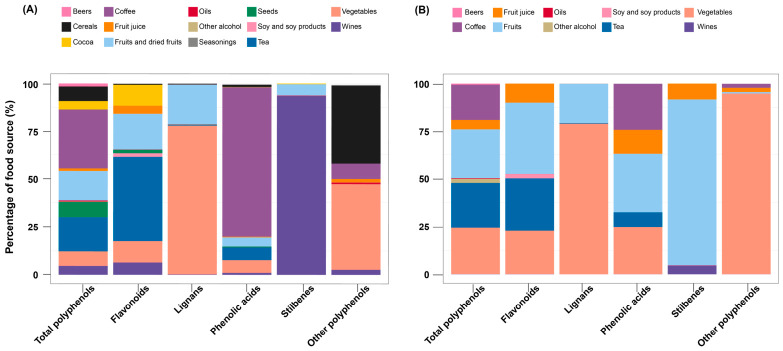
Food sources of dietary polyphenols in (**A**) UK Biobank and (**B**) JACC Study.

Table 2 shows the intake quantities of dietary polyphenols in the two cohorts, which varied between the two cohorts. Compared to the UK Biobank, participants in the JACC Study consumed lower amounts of total polyphenols, flavonoids, lignans, and phenolic acids but higher amounts of stilbenes and other polyphenols. The primary contributors to total polyphenol intake were coffee, fruits, tea, and vegetables in both cohorts. Similarly, flavonoids were mainly derived from tea, fruits, and vegetables, while lignans were primarily obtained from fruits and vegetables across both cohorts. In contrast, some differences were observed for specific subclasses. Phenolic acids were predominantly sourced from coffee in the UK Biobank, whereas in the JACC Study, coffee, fruits, and vegetables contributed substantially. Stilbenes were mainly derived from wines in the UK Biobank but from fruits in the JACC Study. For other polyphenols, cereals and vegetables were the main contributors in the UK Biobank, while vegetables were the predominant source in the JACC Study. Figure 2 illustrates the relative contribution of different food sources to the intake of each polyphenol class within the two cohorts.

**Figure 3:**
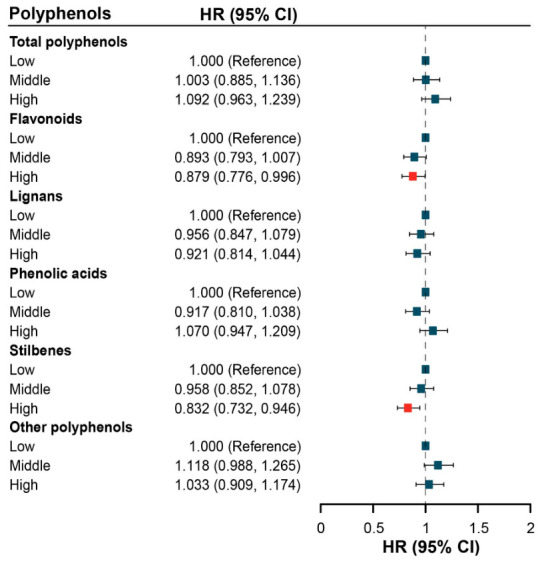
Meta-analysis of the association between dietary polyphenols and lung cancer after full adjustment. Abbreviations: *HR*, hazard ratio; *CI*, confidence interval. A red square indicates a statistically significant association, and a dark blue square indicates a non-significant association.

Table 3 shows the association between dietary polyphenols and lung cancer risk by Cox proportional hazards models. The highest intake of stilbenes was associated with a lower risk of lung cancer in fully adjusted models for both UK Biobank (*HR*: 0.849, 95% *CI*: 0.723–0.996) and JACC Study (*HR*: 0.803, 95% *CI*: 0.648–0.996). In the meta-analysis pooling results from both cohorts, the highest intake of stilbenes (*HR*: 0.832, 95% *CI*: 0.732–0.946) was also associated with a reduced risk of lung cancer (Figure 3). No consistent association was found for other polyphenol subtypes.

**Figure 4:**
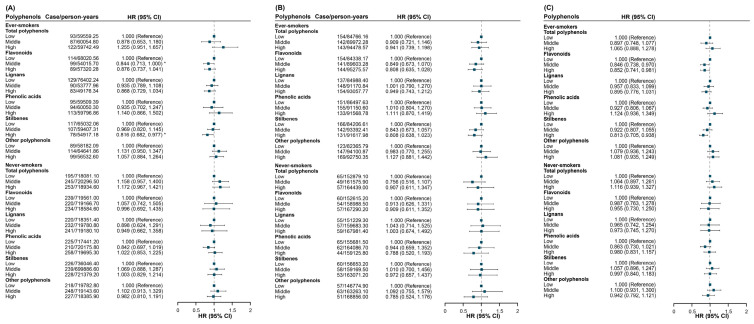
The association between dietary polyphenols and lung cancer stratified by smoking status in (**A**) UK Biobank, (**B**) JACC Study, and (**C**) meta-analysis after full adjustment. * *p* < 0.05. Abbreviations: *HR*, hazard ratio; *CI*, confidence interval. A dark blue square indicates a non-significant association.

In the smoking-status-stratified analysis, significant associations between dietary stilbenes and lung cancer were observed among ever-smokers in UK Biobank (*HR*: 0.816, 95% *CI*: 0.682–0.977) and in the meta-analysis (*HR*: 0.813, 95% *CI*: 0.705–0.938), but not in JACC Study (*HR*: 0.808, 95% *CI*: 0.638–1.023). Additionally, dietary flavonoids were not associated with lung cancer in either cohort, except for a significant association with middle intake levels in the UK Biobank. At the same time, the meta-analysis showed significant associations for both middle intake (*HR*: 0.846, 95% *CI*: 0.738–0.970) and high intake (*HR*: 0.852, 95% *CI*: 0.741–0.981) (Figure 4). No significant associations were found for other polyphenols.

**Figure 5:**
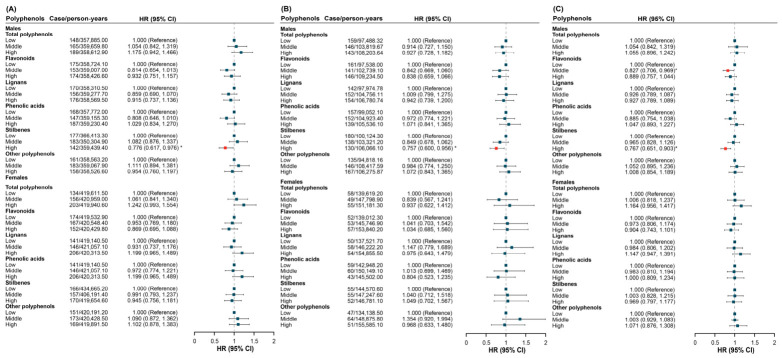
The association between dietary polyphenols and lung cancer stratified by sex in (**A**) UK Biobank, (**B**) JACC Study, and (**C**) meta-analysis after full adjustment. * *p* < 0.05. Abbreviations: *HR*, hazard ratio; *CI*, confidence interval. A red square indicates a statistically significant association, and a dark blue square indicates a non-significant association.

For the sex-stratified analysis, significant associations were found between high stilbene intake and reduced lung cancer risk among males in both cohorts (UK Biobank: *HR*: 0.776, 95% *CI*: 0.617–0.976; JACC Study: *HR*: 0.757, 95% *CI*: 0.600–0.956) and in the pooled results (*HR*: 0.767, 95% *CI*: 0.651–0.903) (Figure 5). Additionally, a significant association between high stilbene intake and reduced lung cancer risk was observed specifically among male ever-smokers in both the UK Biobank (*HR*: 0.722, 95% *CI*: 0.562–0.927), the JACC Study (*HR*: 0.779, 95% *CI*: 0.612–0.990), and the pooled analysis (*HR*: 0.751, 95% *CI*: 0.632–0.893) (Table A1). No significant associations were found for other polyphenols.

**Figure 6:**
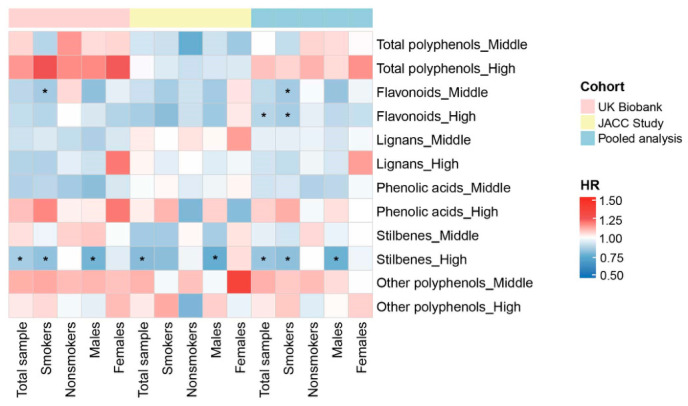
*HR*s for the associations between polyphenols and lung cancer risk among total population and subgroups in different cohorts and pooled analysis. * *p* < 0.05.

The associations between the polyphenols and lung cancer risk among total population and subgroups are presented as a heatmap (Figure 6). Consistent significant associations between dietary stilbenes and lung cancer among total population, smokers, and males can be observed in the individual cohort and pooled analysis. No consistent association was found for other polyphenol subtypes.

**Figure 7:**
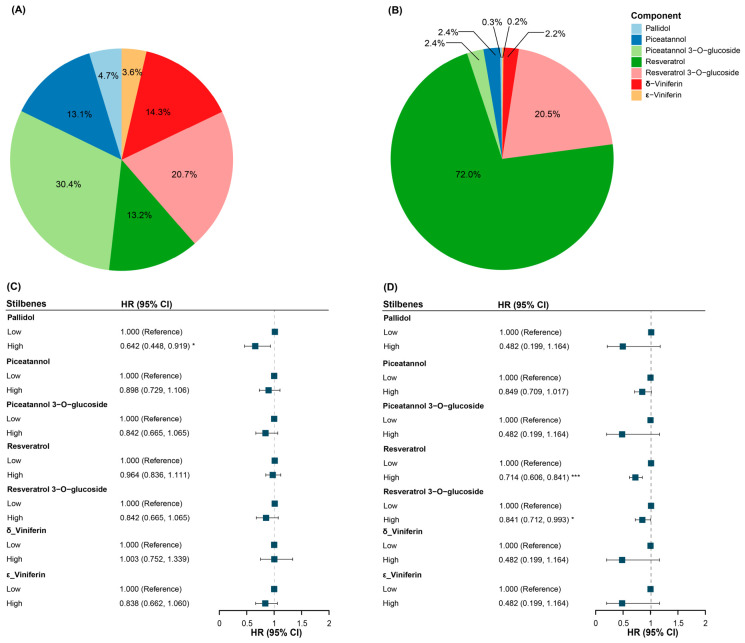
The proportion of each component in stilbenes in (**A**) UK Biobank and (**B**) JACC Study and their associations with lung cancer in (**C**) UK Biobank and (**D**) JACC Study after full adjustment. * *p* < 0.05. *** *p* < 0.001. Pallidol, δ-viniferin, and ε-viniferin are only in wines, and the types of wines in JACC Study were not surveyed, so the results for these three components were the same. Abbreviations: *HR*, hazard ratio; *CI*, confidence interval.

Given the consistent results across the two cohorts and the pooled analysis, we further estimated the proportion of each stilbene compound and their associations with lung cancer to identify the specific compounds responsible for reducing lung cancer risk. In UK Biobank, piceatannol 3-*O*-glucoside (30.4%), resveratrol 3-*O*-glucoside (20.7%), δ-viniferin (14.3%), resveratrol (13.2%), and piceatannol (13.1%) comprised the largest proportions of stilbenes (Figure 7A). However, a significant association with lung cancer was only observed for pallidol (Figure 7C). In the JACC Study, resveratrol (72.0%) and resveratrol 3-*O*-glucoside (20.5%) were the major stilbene compounds, and significant associations were found for both compounds (Figure 7B,D).

**Figure 8:**
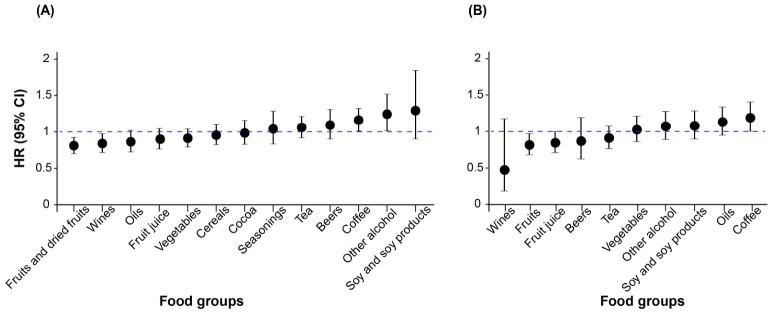
Associations between polyphenol-containing foods and lung cancer in (**A**) UK Biobank and (**B**) JACC Study. For frequently consumed foods, *HR* was the high-median group compared with the low-median group; for infrequently consumed foods, *HR* was the consumption group compared with the non-consumption group. Age, sex, ethnicity (only in UK Biobank), educational level, Townsend deprivation index (only in UK Biobank), marital status (only in JACC Study), active and passive smoking, physical activity, sleep duration, body mass index, family history of lung cancer, energy intake, supplementation, and psychological condition were adjusted. Abbreviations: *HR*, hazard ratio; *CI*, confidence interval.

In the UK Biobank, fruits and dried fruits (*HR*: 0.806, 95% *CI*: 0.703–0.924) and wines (*HR*: 0.836, 95% *CI*: 0.718–0.973) were associated with a lower risk of lung cancer, while coffee (*HR*: 1.156, 95% *CI*: 1.010–1.323) and other alcoholic beverages (*HR*: 1.240, 95% *CI*: 1.014–1.517) were associated with a higher risk. In the JACC Study, wines had the lowest *HR* although there was no significance (*HR*: 0.485, 95% *CI*: 0.201–1.171), fruits (*HR*: 0.817, 95% *CI*: 0.689–0.969) and fruit juice (*HR*: 0.847, 95% *CI*: 0.718–0.999) showed protective effects, while coffee was linked to a higher risk of lung cancer (*HR*: 1.182, 95% *CI*: 1.002–1.396) (Figure 8).

The results of sensitivity analyses indicate that our findings are generally robust (Table A2, Table A3 and Table A4).

## 4. Discussion

Our study showed that, despite different food sources, stilbenes, a subtype of polyphenols, were associated with a lower risk of lung cancer. The inverse association was confined to male ever-smokers. Pooled analyses showed significant associations for flavonoids, although no significance was found within each cohort. Furthermore, the food-based analysis indicated that wines in the UK Biobank and fruits in the JACC Study were the primary dietary sources of stilbenes. These findings suggest that nutrient- and food-based strategies may contribute to lung cancer prevention.

### 4.1. Dietary Polyphenols and Lung Cancer

Lung cancer develops through a complex cascade involving inflammation, oxidative stress, and DNA damage, progressing through stages of initiation, promotion, and malignancy [33,34]. This progress provides an important window for prevention. Current evidence supporting the role of polyphenols in lung cancer prevention comes mainly from in vitro and animal studies. Several mechanisms of polyphenols have been identified, though they vary across polyphenol subtypes. These mechanisms include estrogenic/anti-estrogenic activity, induction of apoptosis, prevention of oxidation, activation of detoxification enzymes, regulation of the immune system, anti-inflammatory effect, and modulation of cellular signaling pathways [35]. Most of these studies have focused on specific compounds, such as resveratrol and curcumin, rather than comprehensively examining all polyphenols [35].

Although no epidemiological studies have specifically examined dietary polyphenols and lung cancer, evidence from other cancers provides relevant implications, with mixed results. For instance, dietary lignans have been shown to prevent breast cancer, while no such effect was observed for total polyphenols in prostate cancer and ovarian cancer [36,37,38]. To our knowledge, this is the first prospective study to examine the association between total and subtypes of dietary polyphenols and lung cancer risk at the population level. We identified the potential preventive effects of dietary stilbenes, especially among male ever-smokers. Although no significant association for flavonoids was found within each cohort, pooled analyses demonstrated significant associations. The potential benefits of these compounds may be attributed to their unique chemical structures, such as unsaturated double bonds and a stable xylene structure, which confer potential for lung cancer prevention [39,40,41].

Therefore, based on our findings and prior research, we hypothesize that the anti-cancer properties of dietary polyphenols depend not only on their structural and functional differences but also on cancer type and population characteristics. Further epidemiological research in diverse populations is urgently needed.

### 4.2. Dietary Stilbenes and Lung Cancer

Our study identified stilbenes as compounds potentially involved in lung cancer prevention, with wines and fruits being their main dietary sources, both associated with a lower risk of lung cancer. Previous mechanistic studies have highlighted the anti-cancer properties of stilbenes. Lung cancer is a complex cascade caused by inflammation, oxidative stress, and DNA damage, progressing through stages of initiation, promotion, and malignancy [33,34]. This progress provides an important window for prevention. Most stilbenes have demonstrated the ability to reduce tumorigenesis across these stages, from initiation and promotion to progression stages of tumorigenesis by enhancing apoptosis, senescence, autophagy, and cell cycle arrest, and inhibiting the invasion and metastasis of cancer cells, due to their antioxidative, anti-inflammatory, antiaging, and chemo-preventive effects [39,42,43].

Several compounds in stilbenes might play important roles against lung cancer. Resveratrol (3,5,4′-trihydroxy-trans-stilbene), the most common natural stilbene, is abundant in fruits such as grapes, red wines, and peanuts [44]. Its anti-inflammatory, antioxidative, proapoptotic, and cell cycle arrest properties enable it to convert free radicals into unreactive compounds [45,46,47]. Our study found the preventive effect of resveratrol and resveratrol 3-O-glucoside in the JACC Study, which might be supported by previous mechanistic studies. These in vitro and in vivo studies have shown that resveratrol acts as a lung cancer chemo-preventive agent by decreasing cancer cell proliferation and inhibiting tumor growth, inducing cell cycle arrest, inducing cell apoptosis, and inhibiting metastasis of lung cancer [48]. In particular, apoptosis induction of primary cancer cells and cancer cells may be the primary mechanism through multiple signaling pathways such as kinases, AKT, and STAT3 [49]. Resveratrol 3-O-glucoside, a glucoside of resveratrol, also known as pieced and polydatin, is probably the most abundant form of resveratrol in nature [50]. It has higher bioavailability and better antioxidant activity due to its resistance to enzymatic oxidation because of the glycosylation of resveratrol [51]. Previous studies provided the mechanistic explanation for our findings, which showed that resveratrol 3-O-glucoside can prevent lung cancer by several pathways, such as by repairing DNA damage, halting cell cycle progression of lung cancer cell line, and inhibiting the NF-kB pathway in non-small-cell lung cancer cells (NSCLC), the most common type of lung cancer [52,53].

We also found an inverse association between dietary pallidol and lung cancer in UK Biobank. Pallidol (C_28_H_22_O_6_), the most active resveratrol dimer, is found in the highest wine concentration. Few studies have investigated its effect on lung cancer. At the same time, a recent in vivo study found that pallidol showed a potential anti-angiogenic effect by inhibiting vascular endothelial growth factor receptor-2 (VEGFR-2) activation and inducing endothelial nitric oxide synthase (eNOS) phosphorylation [54], which might provide a therapy to NSCLC [55].

The differences in the association between stilbene compounds and lung cancer between the two cohorts may be due to varying intake quantities of stilbene compounds from different food sources. In the UK Biobank, wines, the main food sources of stilbenes, were associated with a lower risk of lung cancer, while in the JACC Study, fruits and fruit juice, the primary sources of stilbenes, were associated with a lower risk in food-based analysis. This variation was reflected in the intake quantities of these stilbene compounds: pallidol, predominant in the UK Biobank, is mainly found in wines, while resveratrol and resveratrol 3-O-glucoside, abundant in fruits, comprised the largest proportions of stilbenes in the JACC Study. Additionally, investigation of their associations with lung cancer may be impeded by the small number of participants consuming these compounds and their low intake levels. Therefore, considering these factors and the low bioavailability of stilbenes, we infer that the compounds may only play a preventive role when reaching a certain intake level. From a public health perspective, increasing the intake of stilbene-rich foods, such as wines and fruits, may help prevent lung cancer. Although several mechanistic studies support these findings, epidemiological studies are needed to verify our results.

### 4.3. Dietary Stilbenes and Lung Cancer in Subgroups

After stratifying by smoking status, the protective effect of stilbenes was observed only in ever-smokers, which previous studies might partially support. Epidemiological studies have shown an inverse association between wines, the main source of stilbenes, and the risk of lung cancer among ever-smokers but not never-smokers [56,57]. Similar findings were also reported for fruits, another important food source of stilbenes [10,58]. Consistent with epidemiological studies, mechanistic studies have also shown that polyphenols protect against smoking-induced inflammation-mediated disorders such as lung cancer [59]. Gene mutations may explain this heterogeneity. Ever-smokers and never-smokers with lung cancer may have different and exclusive gene mutations. [60] For example, lung cancers in never-smokers are much more likely to carry activating mutations of the Epidermal Growth Factor Receptor (EGFR), a key oncogenic factor, which is less clearly linked to smoking-related lung cancer [61]. Therefore, genetic differences may influence the response to stilbenes, with their antioxidative properties being more effective among smokers.

In the sex-stratification analysis, the significant association between stilbene intake and lung cancer was only observed among males in both cohorts. Although no similar studies were found, some indirect evidence supports our results. Further analysis confirmed the protective effect of stilbenes specifically among male ever-smokers. The proportion of males among ever-smokers was higher in both cohorts (51.8% in the UK Biobank; 89.5% in the JACC Study), making our sex-stratification results plausible. Previous studies also found that moderate wine intake might lower the risk of lung cancer among men but not women [62]. Additionally, there is also sex heterogeneity in lung cancer, with squamous cell carcinoma more frequent in men and adenocarcinoma in women, along with differences in gene expression, mutations, and polymorphisms between genders [63,64]. These differences may lead to various responses to stilbenes. However, direct evidence in both epidemiological and experimental studies is lacking. Further research focusing on dietary stilbenes and lung cancer, particularly in different subgroups and related mechanisms, is needed.

### 4.4. Biological Plausibility for Stilbenes and Interpretation

It should be noticed that the finding of association between stilbenes and reduced lung cancer risk must be interpreted in light of exposure quantity and pharmacokinetic considerations. In both cohorts, median stilbene intake was extremely low (0.09 mg/day in the UK Biobank and 0.29 mg/day in the JACC Study), whereas median intakes of flavonoids and phenolic acids typically reach one hundred milligrams per day. Pharmacokinetic studies have shown that only relatively large single doses (tens of milligrams or more) produce micromolar plasma concentrations of metabolites. For example, a systematic review reported that the median maximum concentration (Cmax) of polyphenol metabolites from foods was approximately 0.09 µM (90 nmol/L), with substantially higher concentrations observed only after supplementation or high-dose interventions [18]. A 50 mg dose of a flavonoid can reach a Cmax of 1.46 µmol/L, whereas a phenolic acid can reach 0.96 µmol/L [65]. Collectively, these data imply that habitual dietary stilbene intakes (<1 mg/day) are unlikely to yield systemic parent-compound or metabolite concentrations comparable to those of flavonoids or phenolic acids, making a direct pharmacological effect of stilbenes at typical dietary doses biologically implausible.

Therefore, we infer that the observed protective effects for stilbenes may be attributed to these more abundant compounds, such as flavonoids and phenolic acids, rather than trace amounts of stilbenes. Indeed, pooled analyses showed significant associations for flavonoids. Previous epidemiolocal and experimental studies support this interpretation. A recent systematic review and meta-analysis summarizing 20 published papers found that a higher intake of flavonoids was associated with lower risk of lung cancer [66]. This finding is biologically plausible since flavonoids, the largest class of polyphenols, are abundant in both fruits and wines. Numerous studies have demonstrated the mechanisms by which flavonoids may prevent and treat lung cancer, primarily by inhibiting the production of reactive oxygen species (ROS) [67]. This action influences several essential cellular processes, including cell cycle arrest, activation of apoptosis, and modulation of carcinogen-metabolizing enzymes, all directly linked to the inhibition of lung cancer development or progression [68,69].

Similarly, phenolic acids, another abundant class of polyphenols found in wines and fruits, have demonstrated anti-cancer effects through multiple molecular mechanisms [70]. They inhibit tumor cell proliferation and angiogenesis, suppress invasion and metastasis, and promote apoptosis by modulating key signaling pathways such as PI3K/Akt, MAPK, and NF-κB [16,71,72]. In addition, phenolic acids reduce oxidative stress and inflammatory responses by scavenging reactive oxygen species and downregulating COX-2 and cytokine expression, thereby attenuating tumor growth and progression [73].

Taken together, based on our findings and biological plausibility, a more parsimonious explanation is that stilbenes act as markers of polyphenol-rich foods (such as wines and fruits) that contain far greater quantities of flavonoids and phenolic acids. These more abundant classes reach higher plasma concentrations after ordinary dietary intake and are therefore more credible mediators of any protective effect. Furthermore, beverage choices (e.g., wines vs. beers) and fruit consumption are often correlated with socioeconomic status, lifestyle behaviors, and health literacy, which are difficult to fully adjust for, raising the possibility of residual confounding. Until validated by biomarker-based studies or intervention studies that demonstrate that stilbene metabolites at dietary exposure levels exert relevant anti-carcinogenic activity, the observed stilbene association should be considered hypothesis-generating and interpreted with caution.

### 4.5. Strength and Limitations

To our best knowledge, this is the first epidemiological study to investigate the association of various dietary polyphenols and their certain compounds with lung cancer incidence using a pooled analysis method based on two national large-scale cohorts comprising middle-aged and older adults. We also performed stratification analysis by smoking status and sex, to further explore population heterogeneity. Additionally, the results of sensitivity analyses proved the stability of our study.

Despite these strengths, some limitations warrant consideration. First, dietary information relied on dietary recall, which might not accurately capture participants’ typical intake and is susceptible to recall bias. Repeated 24 h recalls were conducted in the UK Biobank, while assessment timing varied across participants and was not standardized by season. In the JACC Study, the FFQ captured habitual intake over the previous year but may have limited precision for estimating specific bioactive compounds such as polyphenols. Moreover, despite standardized and validated portion sizes, regional dietary diversity in Japan may have introduced residual variation not fully captured by the FFQ. Second, there were differences in dietary survey methods and study periods across the two cohorts, potentially affecting estimates of dietary polyphenols, lung cancer incidence, and covariates, thereby making the studies not entirely comparable. These differences also reflect substantial variation in dietary patterns and primary food sources of stilbenes. Despite this, consistent inverse associations between dietary stilbenes and lung cancer risk, particularly among male ever-smokers, were observed in both cohorts, strengthening the generalizability of our findings. Additionally, the FFQ has been shown to provide reasonably valid estimates of nutrient intakes compared to the 24 h dietary recall [74]. Third, the identification of lung cancer relied on hospital admission records, potentially underestimating case numbers. Finally, potential residual confounding cannot be completely ruled out, including genetic predispositions, environmental exposures, and overall dietary patterns, and causality cannot be definitively established due to the observational design. Therefore, further intervention and mechanistic studies are necessary to confirm causal relationships.

## 5. Conclusions

In conclusion, this study provides the first prospective evidence linking dietary stilbenes to a reduced risk of lung cancer. The findings also indicate that consumption of polyphenol-rich foods such as wines and fruits may play an important role in modulating lung cancer risk in the UK and Japan. Although wine contributes polyphenols, it also contains alcohol, a known risk factor for several cancers; therefore, any benefits from wine consumption should be interpreted cautiously. Considering pharmacokinetic properties and biological plausibility, we infer that stilbenes likely serve as markers of polyphenol-rich foods containing substantially greater amounts of flavonoids and phenolic acids. Further biomarker-based and interventional studies are warranted to clarify causality and elucidate the underlying mechanisms, taking into account regional dietary variations.

## Figures and Tables

**Table 1:** Basic characteristics among participants in UK Biobank and JACC Study.

Characteristics	UK Biobank		JACC Study		*p*-Value
	*n* = 177,971		*n* = 57,971		
Sociodemographic characteristics					
Age, years, *median* (*Q*_1_, *Q*_3_)		57.00 (49.00, 62.00)		58.00 (50.00, 65.00)	<0.001
Sex, *n* (%)					<0.001
	Male	82,472 (46.3)	Male	24,325 (42.0)	
	Female	95,499 (53.7)	Female	33,646 (58.0)	
Ethnicity, *n* (%)					
	White	170,304 (95.7)			
	Asian	2619 (1.47)			
	Black	2132 (1.2)			
	Others	2916 (1.64)			
Educational level, *n* (%)					<0.001
	Primary or below	14,723 (8.27)	Primary or below	28,240 (55.9)	
	Secondary	86,380 (48.5)	Secondary	20,328 (40.2)	
	University	76,868 (43.2)	University	1951 (3.9)	
Socioeconomic deprivation status, *median* (*Q*_1_, *Q*_3_)	TDI	−2.32 (−3.73, 0.05)			
Marital status, *n* (%)					
			Married	51,019 (88.0)	
			Bereaved	5013 (8.7)	
			Divorced	1050 (1.8)	
			Single	889 (1.5)	
Lifestyle characteristics					
Active smoking, *n* (%)					<0.001
	Never	101,609 (57.1)	Never	30,362 (62.7)	
	Ever	76,362 (42.9)	Ever	21,609 (37.3)	
Passive smoking, *n* (%)					<0.001
	Never	142,860 (80.3)	Never	7670 (18.3)	
	Ever	35,111 (19.7)	Ever	34,332 (81.7)	
Physical activity, *n* (%)	IPAQ		Sports frequency		
	Low	32,068 (18.0)	Almost never	42,933 (74.1)	
	Moderate	75,940 (42.7)	1~2 h/week	8639 (14.9)	
	High	69,963 (39.3)	>2 h/week	6399 (11.0)	
Sleep duration, *n* (%)					<0.001
	Normal	159,515 (89.6)	Normal	40,961 (70.7)	
	Shorter	7326 (4.1)	Shorter	13,964 (24.1)	
	Longer	11,130 (6.3)	Longer	3046 (5.3)	
Categories of BMI, *n* (%)					
	18.5–24.9 kg/m^2^	65,861 (37.1)	18.5–22.9 kg/m^2^	29,069 (50.1)	
	<18.5 kg/m^2^	966 (0.5)	<18.5 kg/m^2^	3526 (6.1)	
	25 kg/m^2^–29.9 kg/m^2^	74,110 (41.6)	23 kg/m^2^–24.9 kg/m^2^	13,493 (23.3)	
	≥30 kg/m^2^	37,034 (20.8)	≥25 kg/m^2^	11,883 (20.5)	
Family history of lung cancer, *n* (%)					<0.001
	No	159,663 (89.7)	No	50,050 (86.3)	
	Yes	18,308 (10.3)	Yes	7921 (13.7)	
Dietary information					
Energy intake, *median* (*Q*_1_, *Q*_3_)		1984.46 (1608.75, 2420.65)		1453.14 (1204.00, 1753.48)	<0.001
Supplementation, *n* (%)					<0.001
	No	120,568 (67.7)	No	39,724 (68.5)	
	Yes	57,403 (32.3)	Yes	18,247 (31.5)	
Psychological condition					
Mental disorders, *n* (%)	Depressive disorder		Perceived stress		
	No	157,603 (88.6)	No	9901 (17.1)	
	Yes	20,368 (11.4)	Perhaps	35,496 (61.2)	
	Anxiety disorder		Yes	6224 (10.7)	
	No	171,491 (96.4)	Strongly agree	6350 (11.0)	
	Yes	6480 (3.64)			
Follow-up characteristics					
Lung cancer incidence					<0.001
	No	176,976 (99.4)	No	57,361 (98.9)	
	Yes	995 (0.6)	Yes	610 (1.1)	
Follow-up time, years, *median* (*Q*_1_, *Q*_3_)		13.18 (12.60, 13.98)		13.33 (7.46, 18.03)	<0.001

Abbreviations: *Q*_1_, lower quartile; *Q*_3_, upper quartile; TDI, Townsend deprivation index; IPAQ, International Physical Activity Questionnaire; BMI, body mass index.

**Table 2:** Dietary polyphenols among participants in UK Biobank and JACC Study.

Polyphenols	UK Biobank	JACC Study
Total polyphenols, mg/day, *median* (*Q*_1_, *Q*_3_)	2267.34 (1574.50, 3092.92)	1278.53 (616.94, 1892.22)
Flavonoids, mg/day, *median* (*Q*_1_, *Q*_3_)	866.90 (529.81, 1239.29)	672.10 (314.04, 1021.94)
Lignans, mg/day, *median* (*Q*_1_, *Q*_3_)	31.66 (10.55, 80.04)	24.93 (11.33, 39.73)
Phenolic acids, mg/day, *median* (*Q*_1_, *Q*_3_)	1236.24 (491.85, 1924.21)	466.07 (203.80, 732.97)
Stilbenes, mg/day, *median* (*Q*_1_, *Q*_3_)	0.09 (0.01, 1.45)	0.29 (0.05, 0.92)
Other polyphenols, mg/day, *median* (*Q*_1_, *Q*_3_)	56.54 (32.55, 142.44)	60.17 (7.40, 136.97)

Abbreviations: Q_1_, lower quartile; Q_3_, upper quartile.

**Table 3:** Association between dietary polyphenols and lung cancer in UK Biobank and JACC Study.

Polyphenols	Case/Person-Years	Unadjusted Model	Adjusted Model 1	Adjusted Model 2	Adjusted Model 3
Total polyphenols					
Low	277/777,787.20	1.000 (Reference)	1.000 (Reference)	1.000 (Reference)	1.000 (Reference)
Middle	325/780,422.00	1.171 (0.998, 1.375)	1.103 (0.939, 1.296)	1.076 (0.916, 1.264)	1.061 (0.903, 1.248)
High	393/778,459.60	1.419 (1.217, 1.655) ***	1.316 (1.127, 1.536)	1.222 (1.047, 1.426)	1.157 (0.987, 1.355)
Flavonoids					
Low	350/778,261.20	1.000 (Reference)	1.000 (Reference)	1.000 (Reference)	1.000 (Reference)
Middle	321/779,691.10	0.915 (0.787, 1.065)	0.864 (0.743, 1.006)	0.883 (0.758, 1.028)	0.884 (0.759, 1.029)
High	324/778,716.50	0.924 (0.794, 1.075)	0.874 (0.752, 1.017)	0.894 (0.768, 1.040)	0.897 (0.767, 1.048)
Lignans					
Low	343/777,499.00	1.000 (Reference)	1.000 (Reference)	1.000 (Reference)	1.000 (Reference)
Middle	325/779,900.50	0.946 (0.812, 1.100)	0.877 (0.753, 1.022)	0.915 (0.785, 1.066)	0.914 (0.784, 1.066)
High	327/779,269.30	0.952 (0.818, 1.107)	0.832 (0.714, 0.970) *	0.870 (0.746, 1.015)	0.873 (0.747, 1.020)
Phenolic acids					
Low	308/777,141.30	1.000 (Reference)	1.000 (Reference)	1.000 (Reference)	1.000 (Reference)
Middle	293/780,397.90	0.950 (0.809, 1.115)	0.880 (0.749, 1.033)	0.866 (0.737, 1.018)	0.867 (0.738, 1.019)
High	394/779,129.60	1.279 (1.102, 1.485)	1.192 (1.026, 1.384)	1.095 (0.943, 1.272)	1.094 (0.941, 1.272)
Stilbenes					
Low	343/801,078.50	1.000 (Reference)	1.000 (Reference)	1.000 (Reference)	1.000 (Reference)
Middle	345/755,704.90	1.068 (0.919, 1.240)	1.022 (0.880, 1.187)	1.048 (0.902, 1.217)	1.047 (0.899, 1.220)
High	307/779,885.50	0.922 (0.790, 1.075)	0.904 (0.773, 1.056)	0.851 (0.728, 0.995) *	0.849 (0.723, 0.996) *
Other polyphenols					
Low	307/779,626.80	1.000 (Reference)	1.000 (Reference)	1.000 (Reference)	1.000 (Reference)
Middle	360/778,751.10	1.175 (1.009, 1.368)	1.142 (0.980, 1.331)	1.118 (0.959, 1.304)	1.120 (0.958, 1.311)
High	328/778,291.00	1.070 (0.916, 1.250)	1.009 (0.863, 1.180)	1.025 (0.876, 1.199)	1.033 (0.878, 1.215)
JACC Study					
Total polyphenols					
Low	230/236,553.40	1.000 (Reference)	1.000 (Reference)	1.000 (Reference)	1.000 (Reference)
Middle	192/252,286.30	0.776 (0.641, 0.940) **	0.896 (0.739, 1.087)	0.924 (0.761, 1.122)	0.923 (0.759, 1.123)
High	188/259,271.30	0.739 (0.609, 0.897) **	0.932 (0.767, 1.133)	0.978 (0.802, 1.193)	0.990 (0.805, 1.219)
Flavonoids					
Low	237/236,680.50	1.000 (Reference)	1.000 (Reference)	1.000 (Reference)	1.000 (Reference)
Middle	195/248,530.40	0.879 (0.726, 1.063)	0.879 (0.726, 1.063)	0.910 (0.751, 1.102)	0.908 (0.749, 1.102)
High	178/262,900.00	0.798 (0.655, 0.971) *	0.798 (0.655, 0.971) *	0.847 (0.693, 1.036)	0.848 (0.688, 1.044)
Lignans					
Low	216/235,468.70	1.000 (Reference)	1.000 (Reference)	1.000 (Reference)	1.000 (Reference)
Middle	200/250,819.00	0.854 (0.705, 1.036)	1.002 (0.826, 1.216)	1.032 (0.850, 1.253)	1.028 (0.845, 1.252)
High	194/261,823.30	0.787 (0.648, 0.956) *	0.957 (0.787, 1.165)	1.014 (0.832, 1.235)	1.014 (0.824, 1.248)
Phenolic acids					
Low	232/241,654.70	1.000 (Reference)	1.000 (Reference)	1.000 (Reference)	1.000 (Reference)
Middle	203/255,685.50	0.817 (0.677, 0.987) *	0.952 (0.788, 1.150)	0.994 (0.821, 1.203)	0.993 (0.819, 1.205)
High	175/250,770.90	0.718 (0.590, 0.874) ***	0.979 (0.803, 1.195)	1.012 (0.827, 1.239)	1.025 (0.832, 1.263)
Stilbenes					
Low	264/240,711.10	1.000 (Reference)	1.000 (Reference)	1.000 (Reference)	1.000 (Reference)
Middle	207/270,998.50	0.693 (0.578, 0.831) ***	0.821 (0.684, 0.985) *	0.847 (0.706, 1.018)	0.842 (0.700, 1.012)
High	139/236,401.40	0.528 (0.430, 0.648) ***	0.766 (0.622, 0.944) *	0.813 (0.659, 1.002)	0.803 (0.648, 0.996) *
Other polyphenols					
Low	191/228,089.90	1.000 (Reference)	1.000 (Reference)	1.000 (Reference)	1.000 (Reference)
Middle	210/257,866.60	0.946 (0.778, 1.152)	1.069 (0.877, 1.301)	1.115 (0.914, 1.360)	1.115 (0.913, 1.362)
High	209/262,154.50	0.917 (0.753, 1.117)	0.980 (0.804, 1.194)	1.028 (0.843, 1.255)	1.032 (0.838, 1.271)

* *p* < 0.05. ** *p* < 0.01. *** *p* < 0.001. Model 1 adjusted for age, sex, ethnicity (only in UK Biobank), educational level, Townsend deprivation index (only in UK Biobank), and marital status (only in JACC Study). Model 2 adjusted for active and passive smoking, physical activity, sleep duration, body mass index, and family history of lung cancer based on Model 1. Model 3 adjusted for energy intake, supplementation, and psychological condition based on Model 2.

## Data Availability

The data from UK Biobank is available elsewhere (https://www.ukbiobank.ac.uk/), and data from the JACC Study cannot be shared for privacy or ethical reasons.

## Abbreviations

The following abbreviations are used in this manuscript:

*CI*	confidence interval
*HR*	hazard ratio
ICD	International Classification of Diseases
JACC	Japan Collaborative Cohort Study for Evaluation of Cancer Risk
NSCLC	non-small-cell lung cancer cells
*Q*_1_	lower quartile
*Q*_3_	upper quartile
*SD*	standard deviation
TDI	Townsend deprivation index

## Appendix A

### Appendix A.2

**Table A1:** Sex- and smoking-status-specific association between dietary stilbenes and lung cancer.

Stilbenes	Case/Person-Years	Unadjusted Model	Adjusted Model 1	Adjusted Model 2	Adjusted Model 3
Male ever-smoker					
Low	160/171,443.70	1.000 (Reference)	1.000 (Reference)	1.000 (Reference)	1.000 (Reference)
Middle	163/169,843.40	1.029 (0.828, 1.280)	1.022 (0.822, 1.271)	1.021 (0.821, 1.270)	1.032 (0.826, 1.291)
High	111/169,462.20	0.703 (0.552, 0.896) **	0.704 (0.551, 0.900) **	0.714 (0.558, 0.913) **	0.722 (0.562, 0.927) *
Female ever-smoker					
Low	129/161,610.60	1.000 (Reference)	1.000 (Reference)	1.000 (Reference)	1.000 (Reference)
Middle	123/160,039.40	0.964 (0.753, 1.235)	0.908 (0.709, 1.162)	0.914 (0.713, 1.171)	0.901 (0.700, 1.159)
High	120/161,635.60	0.931 (0.726, 1.194)	0.931 (0.724, 1.198)	0.933 (0.724, 1.202)	0.916 (0.706, 1.189)
Male never-smoker					
Low	17/194,969.60	1.000 (Reference)	1.000 (Reference)	1.000 (Reference)	1.000 (Reference)
Middle	26/181,458.10	1.644 (0.892, 3.029)	1.588 (0.862, 2.928)	1.608 (0.872, 2.965)	1.520 (0.814, 2.835)
High	25/188,980.60	1.521 (0.821, 2.816)	1.242 (0.667, 2.311)	1.262 (0.677, 2.351)	1.187 (0.628, 2.242)
Female never-smoker					
Low	37/273,054.50	1.000 (Reference)	1.000 (Reference)	1.000 (Reference)	1.000 (Reference)
Middle	45/244,199.40	1.362 (0.882, 2.104)	1.282 (0.829, 1.981)	1.290 (0.834, 1.994)	1.301 (0.836, 2.024)
High	39/259,971.60	1.111 (0.709, 1.742)	1.028 (0.654, 1.617)	1.045 (0.663, 1.645)	1.055 (0.663, 1.678)
JACC Study					
Male ever-smoker					
Low	159/75,527.32	1.000 (Reference)	1.000 (Reference)	1.000 (Reference)	1.000 (Reference)
Middle	133/82,620.66	0.751 (0.596, 0.945) *	0.851 (0.676, 1.073)	0.855 (0.677, 1.078)	0.846 (0.670, 1.067)
High	127/83,860.82	0.710 (0.562, 0.897) **	0.781 (0.618, 0.987) *	0.795 (0.627, 1.007)	0.779 (0.612, 0.990) *
Female ever-smoker					
Low	5/8504.21	1.000 (Reference)	1.000 (Reference)	1.000 (Reference)	1.000 (Reference)
Middle	9/10,147.97	1.496 (0.501, 4.468)	1.916 (0.581, 6.314)	1.776 (0.532, 5.928)	1.526 (0.444, 5.243)
High	6/8556.04	1.143 (0.348, 3.755)	1.683 (0.448, 6.321)	1.900 (0.489, 7.384)	1.766 (0.433, 7.201)
Male never-smoker					
Low	11/23,406.64	1.000 (Reference)	1.000 (Reference)	1.000 (Reference)	1.000 (Reference)
Middle	9/21,124.34	0.906 (0.375, 2.186)	1.047 (0.430, 2.551)	1.060 (0.430, 2.610)	1.119 (0.452, 2.770)
High	9/22,971.84	0.827 (0.342, 1.997)	0.933 (0.382, 2.279)	0.924 (0.374, 2.284)	0.945 (0.375, 2.387)
Female never-smoker					
Low	50/134,676.10	1.000 (Reference)	1.000 (Reference)	1.000 (Reference)	1.000 (Reference)
Middle	46/137,644.00	0.900 (0.603, 1.343)	0.920 (0.616, 1.374)	0.910 (0.609, 1.360)	0.929 (0.620, 1.392)
High	46/139,071.10	0.869 (0.582, 1.298)	0.947 (0.632, 1.419)	0.924 (0.615, 1.388)	0.972 (0.636, 1.485)
Pooled analysis					
Male ever-smoker					
Low	NA	1.000 (Reference)	1.000 (Reference)	1.000 (Reference)	1.000 (Reference)
Middle	NA	0.887 (0.757, 1.039)	0.938 (0.800, 1.099)	0.9340 (0.801, 1.102)	0.938 (0.799, 1.102)
High	NA	0.707 (0.597, 0.836)	0.743 (0.628, 0.881)	0.755 (0.637, 0.896)	0.751 (0.632, 0.893)
Female ever-smoker					
Low	NA	1.000 (Reference)	1.000 (Reference)	1.000 (Reference)	1.000 (Reference)
Middle	NA	0.985 (0.774, 1.254)	0.936 (0.735, 1.193)	0.939 (0.737, 1.197)	0.920 (0.719, 1.178)
High	NA	0.939 (0.736, 1.198)	0.951 (0.742, 1.217)	0.956 (0.745, 1.226)	0.936 (0.725, 1.210)
Male never-smoker					
Low	NA	1.000 (Reference)	1.000 (Reference)	1.000 (Reference)	1.000 (Reference)
Middle	NA	1.359 (0.820, 2.239)	1.390 (0.839, 2.300)	1.410 (0.850, 2.339)	1.378 (0.824, 2.303)
High	NA	1.246 (0.752, 2.064)	1.131 (0.679, 1.884)	1.142 (0.684, 1.907)	1.103 (0.653, 1.864)
Female never-smoker	NA				
Low	NA	1.000 (Reference)	1.000 (Reference)	1.000 (Reference)	1.000 (Reference)
Middle	NA	1.089 (0.811, 1.461)	1.071 (0.798, 1.439)	1.068 (0.795, 1.435)	1.083 (0.804, 1.460)
High	NA	0.969 (0.718, 1.307)	0.982 (0.726, 1.328)	0.976 (0.721, 1.322)	1.009 (0.738, 1.380)

* *p* < 0.05. ** *p* < 0.01. Model 1 adjusted for age, ethnicity (only in UK Biobank), educational level, Townsend deprivation index (only in UK Biobank), and marital status (only in JACC). Model 2 adjusted for passive smoking, physical activity, sleep duration, body mass index, and family history of lung cancer on the basis of Model 1. Model 3 adjusted for energy intake, supplementation, and psychological condition on the basis of Model 2. NA means not available.

### Appendix A.3

**Table A2:** Association between dietary polyphenols and lung cancer in UK Biobank and JACC Study for two-year landmark analysis.

Polyphenols	Unadjusted Model	Adjusted Model 1	Adjusted Model 2	Adjusted Model 3
Total polyphenols				
Low	1.000 (Reference)	1.000 (Reference)	1.000 (Reference)	1.000 (Reference)
Middle	1.214 (1.027, 1.435) *	1.145 (0.968, 1.355)	1.212 (0.914, 1.608)	1.180 (0.890, 1.565)
High	1.488 (1.268, 1.747) ***	1.383 (1.177, 1.625) ***	1.156 (0.920, 1.452)	1.127 (0.896, 1.416)
Flavonoids				
Low	1.000 (Reference)	1.000 (Reference)	1.000 (Reference)	1.000 (Reference)
Middle	0.882 (0.754, 1.031)	0.833 (0.712, 0.974) *	0.850 (0.727, 0.995) *	0.850 (0.726, 0.995) *
High	0.888 (0.759, 1.038)	0.840 (0.719, 0.983) *	0.859 (0.734, 1.005)	0.859 (0.732, 1.010)
Lignans				
Low	1.000 (Reference)	1.000 (Reference)	1.000 (Reference)	1.000 (Reference)
Middle	0.966 (0.826, 1.130)	0.896 (0.766, 1.050)	0.934 (0.798, 1.094)	0.934 (0.797, 1.094)
High	0.942 (0.804, 1.102)	0.823 (0.701, 0.966)	0.860 (0.732, 1.009)	0.863 (0.733, 1.015)
Phenolic acids				
Low	1.000 (Reference)	1.000 (Reference)	1.000 (Reference)	1.000 (Reference)
Middle	0.963 (0.814, 1.138)	0.893 (0.754, 1.057)	0.878 (0.742, 1.040)	0.880 (0.743, 1.041)
High	1.355 (1.161, 1.582)	1.265 (1.082, 1.478)	1.161 (0.993, 1.356)	1.160 (0.992, 1.356)
Stilbenes				
Low	1.000 (Reference)	1.000 (Reference)	1.000 (Reference)	1.000 (Reference)
Middle	1.061 (0.909, 1.239)	1.017 (0.871, 1.188)	1.043 (0.893, 1.219)	1.042 (0.890, 1.221)
High	0.920 (0.785, 1.079)	0.902 (0.767, 1.060)	0.848 (0.722, 0.998) *	0.847 (0.717, 0.999) *
Other polyphenols				
Low	1.000 (Reference)	1.000 (Reference)	1.000 (Reference)	1.000 (Reference)
Middle	1.179 (1.006, 1.381)	1.148 (0.979, 1.346)	1.123 (0.958, 1.318)	1.126 (0.957, 1.325)
High	1.086 (0.924, 1.276)	1.027 (0.873, 1.208)	1.043 (0.887, 1.227)	1.052 (0.890, 1.244)
JACC Study				
Total polyphenols				
Low	1.000 (Reference)	1.000 (Reference)	1.000 (Reference)	1.000 (Reference)
Middle	0.749 (0.612, 0.918) **	0.863 (0.704, 1.057)	0.888 (0.723, 1.090)	0.885 (0.719, 1.088)
High	0.710 (0.578, 0.871) **	0.890 (0.724, 1.094)	0.930 (0.754, 1.147)	0.937 (0.752, 1.167)
Flavonoids				
Low	1.000 (Reference)	1.000 (Reference)	1.000 (Reference)	1.000 (Reference)
Middle	0.773 (0.633, 0.943) *	0.861 (0.705, 1.052)	0.889 (0.727, 1.088)	0.885 (0.722, 1.085)
High	0.641 (0.521, 0.789) ***	0.751 (0.609, 0.926) *	0.794 (0.641, 0.982) *	0.789 (0.632, 0.985) *
Lignans				
Low	1.000 (Reference)	1.000 (Reference)	1.000 (Reference)	1.000 (Reference)
Middle	0.829 (1.206, 1.677)	0.971 (0.791, 1.191)	0.997 (0.812, 1.224)	0.992 (0.805, 1.221)
High	0.766 (1.306, 0.624)	0.932 (0.758, 1.147)	0.983 (0.798, 1.212)	0.979 (0.786, 1.219)
Phenolic acids				
Low	1.000 (Reference)	1.000 (Reference)	1.000 (Reference)	1.000 (Reference)
Middle	0.786 (0.645, 0.959) *	0.913 (0.748, 1.114)	0.951 (0.778, 1.164)	0.949 (0.774, 1.163)
High	0.683 (0.555, 0.841) ***	0.925 (0.749, 1.141)	0.956 (0.772, 1.185)	0.965 (0.773, 1.203)
Stilbenes				
Low	1.000 (Reference)	1.000 (Reference)	1.000 (Reference)	1.000 (Reference)
Middle	0.694 (0.573, 0.841) ***	0.818 (0.675, 0.992) *	0.843 (0.695, 1.022)	0.834 (0.687, 1.013)
High	0.506 (0.406, 0.630) ***	0.732 (0.586, 0.914) **	0.774 (0.619, 0.968) *	0.762 (0.606, 0.958) *
Other polyphenols				
Low	1.000 (Reference)	1.000 (Reference)	1.000 (Reference)	1.000 (Reference)
Middle	0.909 (0.738, 1.119)	1.021 (0.828, 1.258)	1.062 (0.861, 1.311)	1.060 (0.857, 1.310)
High	0.897 (0.729, 1.103)	0.956 (0.776, 1.177)	0.999 (0.810, 1.232)	0.999 (0.802, 1.244)

* *p* < 0.05. ** *p* < 0.01. *** *p* < 0.001. Model 1 adjusted for age, sex, ethnicity (only in UK Biobank), educational level, Townsend deprivation index (only in UK Biobank), and marital status (only in JACC Study). Model 2 adjusted for active and passive smoking, physical activity, sleep duration, body mass index, and family history of lung cancer on the basis of Model 1. Model 3 adjusted for energy intake, supplementation, and psychological condition on the basis of Model 2.

### Appendix A.4

**Table A3:** Association between dietary polyphenols and lung cancer in UK Biobank and JACC Study without missing covariate value imputation.

Polyphenols	Unadjusted Model	Adjusted Model 1	Adjusted Model 2	Adjusted Model 3
Total polyphenols				
Low	1.000 (Reference)	1.000 (Reference)	1.000 (Reference)	1.000 (Reference)
Middle	1.281 (1.038, 1.581)	1.160 (0.940, 1.433)	1.149 (0.930, 1.419)	1.155 (0.934, 1.429)
High	1.293 (1.048, 1.597)	1.147 (0.928, 1.418)	1.093 (0.884, 1.351)	1.108 (0.892, 1.376)
Flavonoids				
Low	1.000 (Reference)	1.000 (Reference)	1.000 (Reference)	1.000 (Reference)
Middle	0.924 (0.751, 1.137)	0.854 (0.694, 1.051)	0.871 (0.707, 1.072)	0.876 (0.711, 1.079)
High	1.022 (0.835, 1.251)	0.939 (0.767, 1.150)	0.960 (0.784, 1.175)	0.975 (0.792, 1.201)
Lignans				
Low	1.000 (Reference)	1.000 (Reference)	1.000 (Reference)	1.000 (Reference)
Middle	1.138 (0.921, 1.406)	1.021 (0.825, 1.262)	1.053 (0.851, 1.303)	1.060 (0.856, 1.313)
High	1.247 (1.014, 1.534)	1.038 (0.842, 1.280)	1.081 (0.877, 1.334)	1.098 (0.887, 1.358)
Phenolic acids				
Low	1.000 (Reference)	1.000 (Reference)	1.000 (Reference)	1.000 (Reference)
Middle	1.061 (0.863, 1.304)	1.000 (0.814, 1.230)	1.030 (0.837, 1.266)	1.037 (0.841, 1.280)
High	1.036 (0.845, 1.271)	0.956 (0.778, 1.175)	0.919 (0.748, 1.130)	0.925 (0.748, 1.143)
Stilbenes				
Low	1.000 (Reference)	1.000 (Reference)	1.000 (Reference)	1.000 (Reference)
Middle	1.079 (0.882, 1.319)	0.963 (0.786, 1.179)	0.909 (0.742, 1.113)	0.910 (0.742, 1.115)
High	0.917 (0.745, 1.129)	0.812 (0.658, 1.000)	0.807 (0.655, 0.996) *	0.808 (0.655, 0.997) *
Other polyphenols				
Low	1.000 (Reference)	1.000 (Reference)	1.000 (Reference)	1.000 (Reference)
Middle	1.143 (0.929, 1.406)	1.100 (0.893, 1.356)	1.087 (0.882, 1.339)	1.096 (0.886, 1.356)
High	1.125 (0.914, 1.385)	1.050 (0.852, 1.294)	1.064 (0.863, 1.312)	1.081 (0.871, 1.342)
JACC Study				
Total polyphenols				
Low	1.000 (Reference)	1.000 (Reference)	1.000 (Reference)	1.000 (Reference)
Middle	0.800 (0.593, 1.081)	0.900 (0.666, 1.216)	0.924 (0.683, 1.250)	0.912 (0.673, 1.236)
High	0.763 (0.568, 1.023)	0.929 (0.690, 1.249)	0.976 (0.724, 1.315)	0.947 (0.695, 1.292)
Flavonoids				
Low	1.000 (Reference)	1.000 (Reference)	1.000 (Reference)	1.000 (Reference)
Middle	0.830 (0.623, 1.107)	0.877 (0.657, 1.170)	0.910 (0.681, 1.215)	0.890 (0.665, 1.191)
High	0.629 (0.470, 0.843) **	0.689 (0.512, 0.927) *	0.735 (0.545, 0.991) *	0.697 (0.512, 0.950) *
Lignans				
Low	1.000 (Reference)	1.000 (Reference)	1.000 (Reference)	1.000 (Reference)
Middle	0.814 (0.610, 1.086)	0.928 (0.694, 1.240)	0.952 (0.712, 1.273)	0.933 (0.695, 1.251)
High	0.759 (0.569, 1.013)	0.889 (0.663, 1.192)	0.946 (0.704, 1.271)	0.912 (0.670, 1.241)
Phenolic acids				
Low	1.000 (Reference)	1.000 (Reference)	1.000 (Reference)	1.000 (Reference)
Middle	0.868 (0.647, 1.166)	0.991 (0.738, 1.332)	1.033 (0.768, 1.390)	1.024 (0.759, 1.381)
High	0.740 (0.551, 0.994) *	0.999 (0.741, 1.345)	1.031 (0.764, 1.393)	1.014 (0.745, 1.380)
Stilbenes				
Low	1.000 (Reference)	1.000 (Reference)	1.000 (Reference)	1.000 (Reference)
Middle	0.638 (0.487, 0.837) **	0.731 (0.557, 0.959) *	0.754 (0.574, 0.989) *	0.742 (0.565, 0.976) *
High	0.446 (0.332, 0.600) ***	0.619 (0.458, 0.835) **	0.665 (0.492, 0.900) **	0.640 (0.470, 0.871) **
Other polyphenols				
Low	1.000 (Reference)	1.000 (Reference)	1.000 (Reference)	1.000 (Reference)
Middle	1.193 (0.883, 1.613)	1.245 (0.920, 1.685)	1.306 (0.964, 1.769)	1.297 (0.956, 1.759)
High	0.958 (0.695, 1.319)	0.943 (0.684, 1.302)	1.003 (0.726, 1.386)	0.976 (0.699, 1.362)

* *p* < 0.05. ** *p* < 0.01. *** *p* < 0.001. Model 1 adjusted for age, sex, ethnicity (only in UK Biobank), educational level, Townsend deprivation index (only in UK Biobank), and marital status (only in JACC Study). Model 2 adjusted for active and passive smoking, physical activity, sleep duration, body mass index, and family history of lung cancer on the basis of Model 1. Model 3 adjusted for energy intake, supplementation, and psychological condition on the basis of Model 2.

### Appendix A.5

**Table A4:** Association between dietary polyphenols and lung cancer in UK Biobank for participants with at least two dietary surveys.

Polyphenols	Unadjusted Model	Adjusted Model 1	Adjusted Model 2	Adjusted Model 3
Total polyphenols				
Low	1.000 (Reference)	1.000 (Reference)	1.000 (Reference)	1.000 (Reference)
Middle	1.324 (1.069, 1.638) *	1.243 (1.003, 1.540) *	1.263 (0.982, 1.625)	1.262 (0.979, 1.625)
High	1.543 (1.255, 1.898) ***	1.425 (1.158, 1.754) ***	1.252 (0.974, 1.609)	1.248 (0.966, 1.612)
Flavonoids				
Low	1.000 (Reference)	1.000 (Reference)	1.000 (Reference)	1.000 (Reference)
Middle	0.951 (0.774, 1.167)	0.912 (0.742, 1.120)	0.952 (0.745, 1.216)	0.950 (0.743, 1.215)
High	1.108 (0.909, 1.350)	1.054 (0.864, 1.284)	1.050 (0.827, 1.334)	1.049 (0.821, 1.342)
Lignans				
Low	1.000 (Reference)	1.000 (Reference)	1.000 (Reference)	1.000 (Reference)
Middle	0.873 (0.715, 1.067)	0.809 (0.661, 0.990)	0.918 (0.717, 1.174)	0.913 (0.713, 1.170)
High	0.917 (0.752, 1.117)	0.809 (0.663, 0.989)	0.981 (0.771, 1.249)	0.978 (0.766, 1.248)
Phenolic acids				
Low	1.000 (Reference)	1.000 (Reference)	1.000 (Reference)	1.000 (Reference)
Middle	0.998 (0.808, 1.233)	0.923 (0.746, 1.142)	0.897 (0.699, 1.151)	0.893 (0.696, 1.147)
High	1.327 (1.088, 1.617) **	1.226 (1.005, 1.496) *	1.053 (0.829, 1.338)	1.046 (0.823, 1.330)
Stilbenes				
Low	1.000 (Reference)	1.000 (Reference)	1.000 (Reference)	1.000 (Reference)
Middle	0.699 (0.547, 0.893) **	0.772 (0.604, 0.986) *	0.820 (0.655, 1.027)	0.822 (0.656, 1.029)
High	0.466 (0.359, 0.605) ***	0.584 (0.449, 0.761) ***	0.795 (0.637, 0.992) *	0.793 (0.635, 0.990) *
Other polyphenols				
Low	1.000 (Reference)	1.000 (Reference)	1.000 (Reference)	1.000 (Reference)
Middle	1.141 (0.933, 1.396)	1.111 (0.907, 1.360)	1.103 (0.866, 1.405)	1.091 (0.852, 1.396)
High	1.074 (0.876, 1.317)	1.021 (0.831, 1.254)	1.008 (0.788, 1.290)	1.000 (0.776, 1.290)

* *p* < 0.05. ** *p* < 0.01. *** *p* < 0.001. Model 1 adjusted for age, sex, ethnicity, educational level, and Townsend deprivation index. Model 2 adjusted for active and passive smoking, physical activity, sleep duration, body mass index, and family history of lung cancer on the basis of Model 1. Model 3 adjusted for energy intake, supplementation, and psychological condition on the basis of Model 2.

## References

[1] Ferlay J., Soerjomataram I., Dikshit R., Eser S., Mathers C., Rebelo M., Parkin D.M., Forman D., Bray F. (2015). Cancer incidence and mortality worldwide: Sources, methods and major patterns in GLOBOCAN 2012. Int. J. Cancer.

[2] Sung H., Ferlay J., Siegel R.L., Laversanne M., Soerjomataram I., Jemal A., Bray F. (2021). Global Cancer Statistics 2020: GLOBOCAN Estimates of Incidence and Mortality Worldwide for 36 Cancers in 185 Countries. CA Cancer J. Clin..

[3] Chen S., Cao Z., Prettner K., Kuhn M., Yang J., Jiao L., Wang Z., Li W., Geldsetzer P., Bärnighausen T. (2023). Estimates and Projections of the Global Economic Cost of 29 Cancers in 204 Countries and Territories From 2020 to 2050. JAMA Oncol..

[4] Malhotra J., Malvezzi M., Negri E., La Vecchia C., Boffetta P. (2016). Risk factors for lung cancer worldwide. Eur. Respir. J..

[5] Possenti I., Romelli M., Carreras G., Biffi A., Bagnardi V., Specchia C., Gallus S., Lugo A. (2024). Association between second-hand smoke exposure and lung cancer risk in never-smokers: A systematic review and meta-analysis. Eur. Respir. Rev..

[6] World Health Organization (2003). Diet, Nutrition and the Prevention of Chronic Diseases.

[7] Kulathinal S., Karvanen J., Saarela O., Kuulasmaa K. (2007). Case-cohort design in practice—Experiences from the MORGAM Project. Epidemiol. Perspect. Innov..

[8] Yang J., Qian S., Na X., Zhao A. (2023). Association Between Dietary and Supplemental Antioxidants Intake and Lung Cancer Risk: Evidence from a Cancer Screening Trial. Antioxidants.

[9] Wang M., Qin S., Zhang T., Song X., Zhang S. (2015). The effect of fruit and vegetable intake on the development of lung cancer: A meta-analysis of 32 publications and 20 414 cases. Eur. J. Clin. Nutr..

[10] Wang C., Yang T., Guo X.F., Li D. (2019). The Associations of Fruit and Vegetable Intake with Lung Cancer Risk in Participants with Different Smoking Status: A Meta-Analysis of Prospective Cohort Studies. Nutrients.

[11] Chao C. (2007). Associations Between Beer, Wine, and Liquor Consumption and Lung Cancer Risk: A Meta-analysis. Cancer Epidemiol. Biomark. Prev..

[12] Yang J.J., Yu D., Xiang Y.-B., Blot W., White E., Robien K., Sinha R., Park Y., Takata Y., Lazovich D. (2020). Association of Dietary Fiber and Yogurt Consumption with Lung Cancer Risk: A Pooled Analysis. JAMA Oncol..

[13] Leiter A., Veluswamy R.R., Wisnivesky J.P. (2023). The global burden of lung cancer: Current status and future trends. Nat. Rev. Clin. Oncol..

[14] Kim K.H., Ki M.-R., Min K.H., Pack S.P. (2023). Advanced Delivery System of Polyphenols for Effective Cancer Prevention and Therapy. Antioxidants.

[15] Ali M., Benfante V., Stefano A., Yezzi A., Di Raimondo D., Tuttolomondo A., Comelli A. (2023). Anti-Arthritic and Anti-Cancer Activities of Polyphenols: A Review of the Most Recent In Vitro Assays. Life.

[16] Anantharaju P.G., Gowda P.C., Vimalambike M.G., Madhunapantula S.V. (2016). An overview on the role of dietary phenolics for the treatment of cancers. Nutr. J..

[17] Behl T., Rana T., Alotaibi G.H., Shamsuzzaman M., Naqvi M., Sehgal A., Singh S., Sharma N., Almoshari Y., Abdellatif A.A.H. (2022). Polyphenols inhibiting MAPK signalling pathway mediated oxidative stress and inflammation in depression. Biomed. Pharmacother..

[18] Rothwell J.A., Urpi-Sarda M., Boto-Ordoñez M., Llorach R., Farran-Codina A., Barupal D.K., Neveu V., Manach C., Andres-Lacueva C., Scalbert A. (2016). Systematic analysis of the polyphenol metabolome using the Phenol-Explorer database. Mol. Nutr. Food Res..

[19] Addissouky T.A., El Sayed I.E.T., Ali M.M.A., Wang Y., El Baz A., Elarabany N., Khalil A.A. (2024). Oxidative stress and inflammation: Elucidating mechanisms of smoking-attributable pathology for therapeutic targeting. Bull. Natl. Res. Cent..

[20] Jackson S.S., Marks M.A., Katki H.A., Cook M.B., Hyun N., Freedman N.D., Kahle L.L., Castle P.E., Graubard B.I., Chaturvedi A.K. (2022). Sex disparities in the incidence of 21 cancer types: Quantification of the contribution of risk factors. Cancer.

[21] Kiyohara C., Ohno Y. (2010). Sex differences in lung cancer susceptibility: A review. Gend. Med..

[22] Sudlow C., Gallacher J., Allen N., Beral V., Burton P., Danesh J., Downey P., Elliott P., Green J., Landray M. (2015). UK biobank: An open access resource for identifying the causes of a wide range of complex diseases of middle and old age. PLoS Med..

[23] Mendez M.A., Popkin B.M., Buckland G., Schroder H., Amiano P., Barricarte A., Huerta J.M., Quirós J.R., Sánchez M.J., González C.A. (2011). Alternative methods of accounting for underreporting and overreporting when measuring dietary intake-obesity relations. Am. J. Epidemiol..

[24] Tamakoshi A., Ozasa K., Fujino Y., Suzuki K., Sakata K., Mori M., Kikuchi S., Iso H., Sakauchi F., Motohashi Y. (2013). Cohort profile of the Japan Collaborative Cohort Study at final follow-up. J. Epidemiol..

[25] Tamakoshi A. (2007). Overview of the Japan Collaborative Cohort Study for Evaluation of Cancer (JACC). Asian Pac. J. Cancer Prev. APJCP.

[26] Liu B., Young H., Crowe F.L., Benson V.S., Spencer E.A., Key T.J., Appleby P.N., Beral V. (2011). Development and evaluation of the Oxford WebQ, a low-cost, web-based method for assessment of previous 24 h dietary intakes in large-scale prospective studies. Public Health Nutr..

[27] Royal Society of Chemistry (2014). McCance and Widdowson’s the Composition of Foods.

[28] Date C., Fukui M., Yamamoto A., Wakai K., Ozeki A., Motohashi Y., Adachi C., Okamoto N., Kurosawa M., Tokudome Y. (2005). Reproducibility and validity of a self-administered food frequency questionnaire used in the JACC study. J. Epidemiol..

[29] Hagiwara K. (2001). Standard Tables of Food Composition in Japan Fifth Revised Edition. J. Integr. Study Diet. Habits.

[30] Rothwell J.A., Perez-Jimenez J., Neveu V., Medina-Remón A., M’Hiri N., García-Lobato P., Manach C., Knox C., Eisner R., Wishart D.S. (2013). Phenol-Explorer 3.0: A major update of the Phenol-Explorer database to incorporate data on the effects of food processing on polyphenol content. Database J. Biol. Databases Curation.

[31] Davies M.P.A., Sato T., Ashoor H., Hou L., Liloglou T., Yang R., Field J.K. (2023). Plasma protein biomarkers for early prediction of lung cancer. eBioMedicine.

[32] van Buuren S., Groothuis-Oudshoorn K. (2011). mice: Multivariate Imputation by Chained Equations in R. J. Stat. Softw..

[33] Barnes J.L., Zubair M., John K., Poirier M.C., Martin F.L. (2018). Carcinogens and DNA damage. Biochem. Soc. Trans..

[34] Reuter S., Gupta S.C., Chaturvedi M.M., Aggarwal B.B. (2010). Oxidative stress, inflammation, and cancer: How are they linked?. Free Radic. Biol. Med..

[35] Pandey K.B., Rizvi S.I. (2009). Plant polyphenols as dietary antioxidants in human health and disease. Oxidative Med. Cell. Longev..

[36] Bahrami A., Makiabadi E., Jalali S., Heidari Z., Assadi M., Rashidkhani B. (2021). Dietary Intake of Polyphenols and the Risk of Breast Cancer: A Case-Control Study. Clin. Nutr. Res..

[37] Almanza-Aguilera E., Guiñón-Fort D., Perez-Cornago A., Martínez-Huélamo M., Andrés-Lacueva C., Tjønneland A., Eriksen A.K., Katzke V., Bajracharya R., Schulze M.B. (2023). Intake of the Total, Classes, and Subclasses of (Poly)Phenols and Risk of Prostate Cancer: A Prospective Analysis of the EPIC Study. Cancers.

[38] Londoño C., Cayssials V., de Villasante I., Crous-Bou M., Scalbert A., Weiderpass E., Agudo A., Tjønneland A., Olsen A., Overvad K. (2021). Polyphenol Intake and Epithelial Ovarian Cancer Risk in the European Prospective Investigation into Cancer and Nutrition (EPIC) Study. Antioxidants.

[39] Sirerol J.A., Rodríguez M.L., Mena S., Asensi M.A., Estrela J.M., Ortega A.L. (2016). Role of Natural Stilbenes in the Prevention of Cancer. Oxidative Med. Cell. Longev..

[40] Tian J., Jin L., Liu H., Hua Z. (2023). Stilbenes: A promising small molecule modulator for epigenetic regulation in human diseases. Front. Pharmacol..

[41] Kumar S., Pandey A.K. (2013). Chemistry and biological activities of flavonoids: An overview. Sci. World J..

[42] Reinisalo M., Kårlund A., Koskela A., Kaarniranta K., Karjalainen R.O. (2015). Polyphenol Stilbenes: Molecular Mechanisms of Defence against Oxidative Stress and Aging-Related Diseases. Oxidative Med. Cell. Longev..

[43] Lee Y.H., Chen Y.Y., Yeh Y.L., Wang Y.J., Chen R.J. (2019). Stilbene Compounds Inhibit Tumor Growth by the Induction of Cellular Senescence and the Inhibition of Telomerase Activity. Int. J. Mol. Sci..

[44] Rivière C., Pawlus A.D., Mérillon J.M. (2012). Natural stilbenoids: Distribution in the plant kingdom and chemotaxonomic interest in Vitaceae. Nat. Prod. Rep..

[45] Donnelly L.E., Newton R., Kennedy G.E., Fenwick P.S., Leung R.H., Ito K., Russell R.E., Barnes P.J. (2004). Anti-inflammatory effects of resveratrol in lung epithelial cells: Molecular mechanisms. Am. J. Physiol. Lung Cell. Mol. Physiol..

[46] Albuquerque R.V., Malcher N.S., Amado L.L., Coleman M.D., dos Santos D.C., Borges R.S., Valente S.A.S., Valente V.C., Monteiro M.C. (2015). In Vitro Protective Effect and Antioxidant Mechanism of Resveratrol Induced by Dapsone Hydroxylamine in Human Cells. PLoS ONE.

[47] Benitez D.A., Pozo-Guisado E., Alvarez-Barrientos A., Fernandez-Salguero P.M., Castellón E.A. (2007). Mechanisms involved in resveratrol-induced apoptosis and cell cycle arrest in prostate cancer-derived cell lines. J. Androl..

[48] Yousef M., Vlachogiannis I.A., Tsiani E. (2017). Effects of Resveratrol against Lung Cancer: In Vitro and In Vivo Studies. Nutrients.

[49] Briguglio G., Costa C., Pollicino M., Giambò F., Catania S., Fenga C. (2020). Polyphenols in cancer prevention: New insights (Review). Int. J. Funct. Nutr..

[50] Şöhretoğlu D., Baran M.Y., Arroo R., Kuruüzüm-Uz A. (2018). Recent advances in chemistry, therapeutic properties and sources of polydatin. Phytochem. Rev..

[51] Wang H.L., Gao J.P., Han Y.L., Xu X., Wu R., Gao Y., Cui X.H. (2015). Comparative studies of polydatin and resveratrol on mutual transformation and antioxidative effect in vivo. Phytomedicine.

[52] Verma N., Tiku A.B. (2022). Polydatin-Induced Direct and Bystander Effects in A549 Lung Cancer Cell Line. Nutr. Cancer.

[53] Zou J., Yang Y., Yang Y., Liu X. (2018). Polydatin suppresses proliferation and metastasis of non-small cell lung cancer cells by inhibiting NLRP3 inflammasome activation via NF-κB pathway. Biomed. Pharmacother..

[54] Fernandez-Cruz E., Cerezo A.B., Cantos-Villar E., Richard T., Troncoso A.M., Garcia-Parrilla M.C. (2019). Inhibition of VEGFR-2 Phosphorylation and Effects on Downstream Signaling Pathways in Cultivated Human Endothelial Cells by Stilbenes from *Vitis* spp.. J. Agric. Food Chem..

[55] Daum S., Hagen H., Naismith E., Wolf D., Pircher A. (2020). The Role of Anti-angiogenesis in the Treatment Landscape of Non-small Cell Lung Cancer—New Combinational Approaches and Strategies of Neovessel Inhibition. Front. Cell Dev. Biol..

[56] Chao C., Slezak J.M., Caan B.J., Quinn V.P. (2008). Alcoholic Beverage Intake and Risk of Lung Cancer: The California Men’s Health Study. Cancer Epidemiol. Biomark. Prev..

[57] Bagnardi V., Rota M., Botteri E., Scotti L., Jenab M., Bellocco R., Tramacere I., Pelucchi C., Negri E., La Vecchia C. (2011). Alcohol consumption and lung cancer risk in never smokers: A meta-analysis. Ann. Oncol..

[58] Vieira A.R., Abar L., Vingeliene S., Chan D.S.M., Aune D., Navarro-Rosenblatt D., Stevens C., Greenwood D., Norat T. (2016). Fruits, vegetables and lung cancer risk: A systematic review and meta-analysis. Ann. Oncol..

[59] Rudrapal M., Maji S., Prajapati S.K., Kesharwani P., Deb P.K., Khan J., Mohamed Ismail R., Kankate R.S., Sahoo R.K., Khairnar S.J. (2022). Protective Effects of Diets Rich in Polyphenols in Cigarette Smoke (CS)-Induced Oxidative Damages and Associated Health Implications. Antioxidants.

[60] Kuśnierczyk P. (2023). Genetic differences between smokers and never-smokers with lung cancer. Front. Immunol..

[61] Rudin C.M., Avila-Tang E., Harris C.C., Herman J.G., Hirsch F.R., Pao W., Schwartz A.G., Vahakangas K.H., Samet J.M. (2009). Lung cancer in never smokers: Molecular profiles and therapeutic implications. Clin. Cancer Res..

[62] Freudenheim J.L., Ritz J., Smith-Warner S.A., Albanes D., Bandera E.V., van den Brandt P.A., Colditz G., Feskanich D., Goldbohm R.A., Harnack L. (2005). Alcohol consumption and risk of lung cancer: A pooled analysis of cohort studies. Am. J. Clin. Nutr..

[63] Conforti F., Pala L., Bagnardi V., Viale G., De Pas T., Pagan E., Pennacchioli E., Cocorocchio E., Ferrucci P.F., De Marinis F. (2019). Sex-Based Heterogeneity in Response to Lung Cancer Immunotherapy: A Systematic Review and Meta-Analysis. J. Natl. Cancer Inst..

[64] Frega S., Dal Maso A., Ferro A., Bonanno L., Conte P., Pasello G. (2019). Heterogeneous tumor features and treatment outcome between males and females with lung cancer (LC): Do gender and sex matter?. Crit. Rev. Oncol./Hematol..

[65] Manach C., Williamson G., Morand C., Scalbert A., Rémésy C. (2005). Bioavailability and bioefficacy of polyphenols in humans. I. Review of 97 bioavailability studies. Am. J. Clin. Nutr..

[66] Rostampour K., Alipour K., Mirjalili F., Forootani B., Yekrang Safakar H., Beigrezaei S., Forbes S.C., Salehi-Abargouei A. (2025). Dietary Flavonoids and Lung Cancer: A GRADE-Assessed Systematic Review and Meta-Analysis of Observational Studies. Nutr. Cancer.

[67] ArulJothi K.N., Kumaran K., Senthil S., Nidhu A.B., Munaff N., Janitri V.B., Kirubakaran R., Singh S.K., Gupt G., Dua K. (2022). Implications of reactive oxygen species in lung cancer and exploiting it for therapeutic interventions. Med. Oncol..

[68] Braicu C., Mehterov N., Vladimirov B., Sarafian V., Nabavi S.M., Atanasov A.G., Berindan-Neagoe I. (2017). Nutrigenomics in cancer: Revisiting the effects of natural compounds. Semin. Cancer Biol..

[69] Khan N., Mukhtar H. (2015). Dietary agents for prevention and treatment of lung cancer. Cancer Lett..

[70] Kumar N., Goel N. (2019). Phenolic acids: Natural versatile molecules with promising therapeutic applications. Biotechnol. Rep..

[71] Bao X., Li W., Jia R., Meng D., Zhang H., Xia L. (2023). Molecular mechanism of ferulic acid and its derivatives in tumor progression. Pharmacol. Rep..

[72] Tsao S.M., Hsia T.C., Yin M.C. (2014). Protocatechuic acid inhibits lung cancer cells by modulating FAK, MAPK, and NF-κB pathways. Nutr. Cancer.

[73] Liu W., Cui X., Zhong Y., Ma R., Liu B., Xia Y. (2023). Phenolic metabolites as therapeutic in inflammation and neoplasms: Molecular pathways explaining their efficacy. Pharmacol. Res..

[74] Procter-Gray E., Olendzki B., Kane K., Churchill L., Hayes R.B., Aguirre A., Kang H.J., Li W. (2017). Comparison of Dietary Quality Assessment Using Food Frequency Questionnaire and 24-hour-recalls in Older Men and Women. AIMS Public Health.

